# Scanner-Assisted CO_2_ Laser Fissurectomy: A Pilot Study

**DOI:** 10.3389/fsurg.2021.799607

**Published:** 2021-12-28

**Authors:** Iacopo Giani, Tommaso Cioppa, Chiara Linari, Filippo Caminati, Paolo Dreoni, Gianni Rossi, Cinzia Tanda, Giuseppina Talamo, Federico Bettazzi, Alessandra Aprile, Silvia Grassi, Antonella Pede, Luca Giannoni, Claudio Elbetti

**Affiliations:** ^1^SOSD Proctologia, USL Toscana Centro, Florence, Italy; ^2^Department of CRP (Clinical Research and Practice), El.En. Group, Florence, Italy

**Keywords:** scanner-assisted CO_2_ laser, fissurectomy, chronic anal fissure, faecal incontinence, internal sphincterotomy, wound healing, functional healing, low pain intensity

## Abstract

**Introduction:** Surgery for chronic anal fissure is challenging for every proctologist. Solving the pain by guaranteeing rapid and effective healing is the objective, but what is the price to pay today in functional terms? Though this result is nowadays partially achievable through interventions that include the execution of an internal sphincterotomy among the procedures, it is necessary to underline the high rate of patients who can present faecal incontinence. The aim of this study is to explore the effectiveness of scanner-assisted CO_2_ laser fissurectomy.

**Methods:** From April 2021 to September 2021, all consecutive patients who affected by chronic anal fissure suitable for surgery, meeting the inclusion and exclusion criteria, were evaluated. All planned data were recorded before surgery, then at 24 h, 1 week, and 1 month follow-up. A scanner-assisted CO_2_ laser was used in this study to achieve a smooth and dried wound with a minimal tissue thermal damage, to ensure good postsurgical pain control, rapid and functional, elastic and stable healing, and to prevent potential relapses. Paracetamol 1 g every 8 h was prescribed for the first 24 h and then continued according to each patient's need. Ketorolac 15 mg was prescribed as rescue.

**Results:** Mean pain intensity ≤3, considered as the principal endpoint, was recorded in 26 out of the 29 patients who enrolled in the study with a final success rate of 89.7% at 1-month follow-up. Pain and anal itching showed a statistically significant reduction while bleeding, burning, and maximum pain, and REALIS score showed a reduction too at the end of the follow-up period. Reepithelisation proved to be extremely fast and effective: 22 of 29 (75.9%) showed a complete healing and 5 showed a partial reepithelisation at 1-month follow-up.

**Discussion:** Outcomes of this study showed that it is undoubtedly necessary to change the surgical approach in case of anal fissure. The internal sphincterotomy procedure must be most of all questioned, where the availability of cutting-edge technological tools must be avoided and offered only in selected cases. Scanner-assisted CO_2_ laser showed great results in terms of pain control and wound healing, secondary to an extremely precise ablation, vaporisation, and debridement procedures with minimal lateral thermal damage.

## Introduction

Chronic anal fissure usually causes recurrent pain mostly at defecation and bleeding per annum which negatively affect patients' life quality (1).

Sometimes, it can worsen and evolve into a perianal abscess or fistula in anus and this helps to reinforce the indication to a surgical resolution.

Anal fissure has a widespread diffusion and its incidence is 10–15% among all proctologic consultations (2).

An epidemiologic study underlined that United States count approximately 3,42,000 new anal fissure cases per year where the main affected are middle-aged and younger people with an equal male to female ratio (3).

The exact aetiology is still debated but usually the trauma of the anoderm derived from hard stool passage is considered the main cause followed by chemical irritation due to diarrhoea, postsurgical rigid and retracted scars, and anal intercourse too (4, 5).

This painful wound causes a reactive internal sphincter hypertone and the increase in the resting anal pressure.

Several pharmacological agents have been studied first to directly reduce anal pressure and/or indirectly control anal fissure pain and second both to improve the blood supply to the anal fissure and to facilitate healing (6).

A chronic anal fissure is diagnosed when anal fissure and related symptoms persist not <6 weeks and are associated with the presence of visible transverse internal anal sphincter fibres, sentinel skin tag, anal papillae, anal polyp, and indurated margins (7, 8).

It is sustained by local recurrent traumas, inadequate or failure to adhere to therapies and comorbidities.

When medical treatments fail, surgery is the only solution.

Diathermy fissurectomy is the surgical procedure for removing this chronic longitudinal tear of the anus by eliminating the fibrotic tissue, the sentinel skin tags, and anal polyp. If on the one hand, it is able to remove the fibrotic tissue, on the other hand, the surgical wound results to be very painful. Fissurectomy can be performed alone though it is usually associated with lateral internal sphincterotomy.

Despite the high incidence of anal incontinence (faecal incontinence 1:200, permanent flatus incontinence 1:20) after internal sphincterotomy (9–11), it still remains the gold standard to reduce postoperative pain and allow wound healing.

To avoid the high rate of incontinence secondary to internal sphincterotomy and at the same time ensure good postsurgical pain control and a rapid surgical wound healing too, we explored the effectiveness of scanner-assisted CO_2_ laser technology, already widely used in speciality such as colposcopy, with a similar approach.

CO_2_ laser technology is well known in various surgery fields, from the late 70s. Its wavelength of 10.600 nm is entirely absorbed by water, thus making this laser undoubtedly the best surgical one, due to its excellent characteristics of tissue interaction.

Nevertheless, improvements in CO_2_ laser technology have brought to sources excited with radiofrequency (so-called Ultrapulsed) and the introduction of tools such as surgical scanners, in association with focusing handpieces and high precision microspot micromanipulators coupled to surgical colposcopes or high-definition cameras, which allow to overcome the results of first CO_2_ laser generation. Scanners allow to move a micrometric focused spot on the tissue in an extremely fast (up to 1/1,000,000 of a second of prevalence or “dwell time”) and precise manner, reproducing predefined shapes of cutting and plane ablation. By this way, surgical procedures are extremely selective, and the surrounding healthy tissues are not thermally damaged. Furthermore, coagulation feature of the laser can be improved when needed by simply adapting scanning and emission mode settings.

In general, laser surgery is a minimally invasive procedure that reduces hospitalisation time, decreases the postoperative pain, oedema, and discomfort, and results in fewer complications, faster and more functional wound healing (12, 13).

The aim of this study is to overcome the limits of the current surgery for chronic anal fissure in terms of postoperative pain, pain and other symptom resolutions, healing time, incontinence rate, and patient's satisfaction with respect to safety and reproducibility.

## Methods

From April 2021 to September 2021, all consecutive patients who arrived at the clinics of SOSD Proctologia (USL Toscana Centro-Firenze) and affected by chronic anal fissure (7, 8) suitable for surgery were evaluated.

We planned a strict selection to obtain homogeneous group of patients: we considered only patients complaining anal pain secondary to a single chronic anal fissure unresponsive to medical therapies, ASA 1 and 2 only and Lee index <1 (14), who are able to understand all medical instructions and to adhere to our perioperative protocol.

We excluded those patients with concomitant perianal abscess or fistula in anus or any anal disease or previous proctologic surgeries, with history of radiotherapy, pregnancy, age below 18 years, Crohn's disease, constipation requiring manual manoeuvres during evacuation, anal neoplasms, human immunodeficiency virus infection, faecal incontinence, proctitis, severe systemic diseases, uncontrolled comorbidities as diabetes, kidney failure, and anticoagulant therapy.

All participants provided with verbal and written informed consent either to surgery or to participation in the study.

All patients underwent a complete medical history, clinical evaluation, proctologic physical examination, anoscopy and endoanal ultrasound. All data were collected at different time, preoperative (T0), at 24 h (T1), then at 1 week (T2), and finally at 1-month follow-up (T3), and recorded in a prospective maintained database.

All data collected are reported in Table 1.

**Table 1:** Data collection.

	**T0 Pre Operative**	**T1** **24 h**	**T2** **1 week**	**T3** **1 month**
Anal fissure position (12 o'clock position for anterior and 6 for posterior)	√	–	–	–
Sentinel skin tag	√	–	–	–
Sentinel anal polyp	√	–	–	–
Pain (yes-no)	√	√	√	√
Bleeding (yes-no)	√	√	√	√
Anal itching (yes-no)	√	√	√	√
Burning (yes-no)	√	√	√	√
Constipation (yes-no)	√	√	√	√
Diarrhoea (yes-no)	√	√	√	√
Anal intercourse (yes-no)	√	√	√	√
Duration of symptoms (months)	√	–	–	–
REALISE score	√	–	√	√
Mean pain (VAS 0–10) (Success VAS ≤3)	√	√	√	√
Maximum pain (VAS 0–10)	√	√	√	√
Maximum pain duration 1-Within 10 min 2-Between 10 and 30 min 3-Between 30 and 60 min 4-More then 60 min	√	√	√	√
Anal digital exploration 0-not painful 1-painful 2-impossible	√	–	√	√
Post operative data records - Number of painkiller days - Compliance with anal cream application (yes-no) - Complications (descriptive) - Re intervention (yes-no) - Faecal incontinence	–	√	√	√
- Patient satisfaction (VAS 0–10)	–	–	–	√
Surgeons satisfaction (VAS 0–10)		√	–	–
Degree of reepithelisation of the post surgical fissure 0-deep fissure still present 1-superficial fissure 2-partial reepithelisation 3-complete healing and reepithelisation	–	√	√	√

### Endpoints

The principal endpoint was mean anal pain intensity ≤3 (VAS 0–10).

Anal pain intensity and symptom intensity were measured by means of a 10-point visual analogue scale (VAS) (15).

The following secondary endpoints considered were as follows: maximum pain intensity (VAS 0–10), maximum pain duration (1—within 10 min, 2—between 10 and 30 min, 3—between 30 and 60 min, and 4—more than 60 min), days of painkiller intake, specific symptoms, and REALISE score (16).

Then, we focused on other secondary endpoints: the proportion of patients healed at 1 week and then at 1-month follow-up and graded according to a previous published degree of reepithelisation scale (0—deep fissure still present, 1—superficial fissure, 2—partial reepithelisation, 3—complete healing and reepithelisation); by the end of fissurectomy, all patients were found to be at the lowest grade (17).

Finally, we also considered patient's satisfaction at 1 month and surgeons' satisfaction (about surgery), and these were recorded through a VAS 0–10 scale.

Short-term complications were recorded; reoperation and discharge within 2 h were evaluated too.

### Withdrawal Criteria

Failure to follow the protocol, further surgery during the follow-up, patient request.

### Perioperative Protocol

Patients were instructed to correct constipation already before surgery by taking stool softeners and sticking to a diet rich in fibres and fluids.

After surgery, all patients received written instruction thoroughly explained before discharge.

Surgical wound protection protocol required patients to apply a 3% sucralfate cream (18–20) (Emoflon™- Servier Italia S.p.A.) circumferentially up to 1–2 cm inside the anus with the tip of a finger every 12 h and to daily warm sitz bath for the entire period of the study (21, 22).

Paracetamol 1 g every 8 h was prescribed for the first 24 h and then continued according to each patient's need. Ketorolac 15 mg was prescribed as rescue.

### Treatment

All patients were treated under local anaesthesia (ropivacaine 10 mg/ml, ranging from 5 to 10 ml) performed directly on anal fissure with a 25-G needle, in an ambulatory setting with a planned discharge time of 2 h.

**Figure 1:**
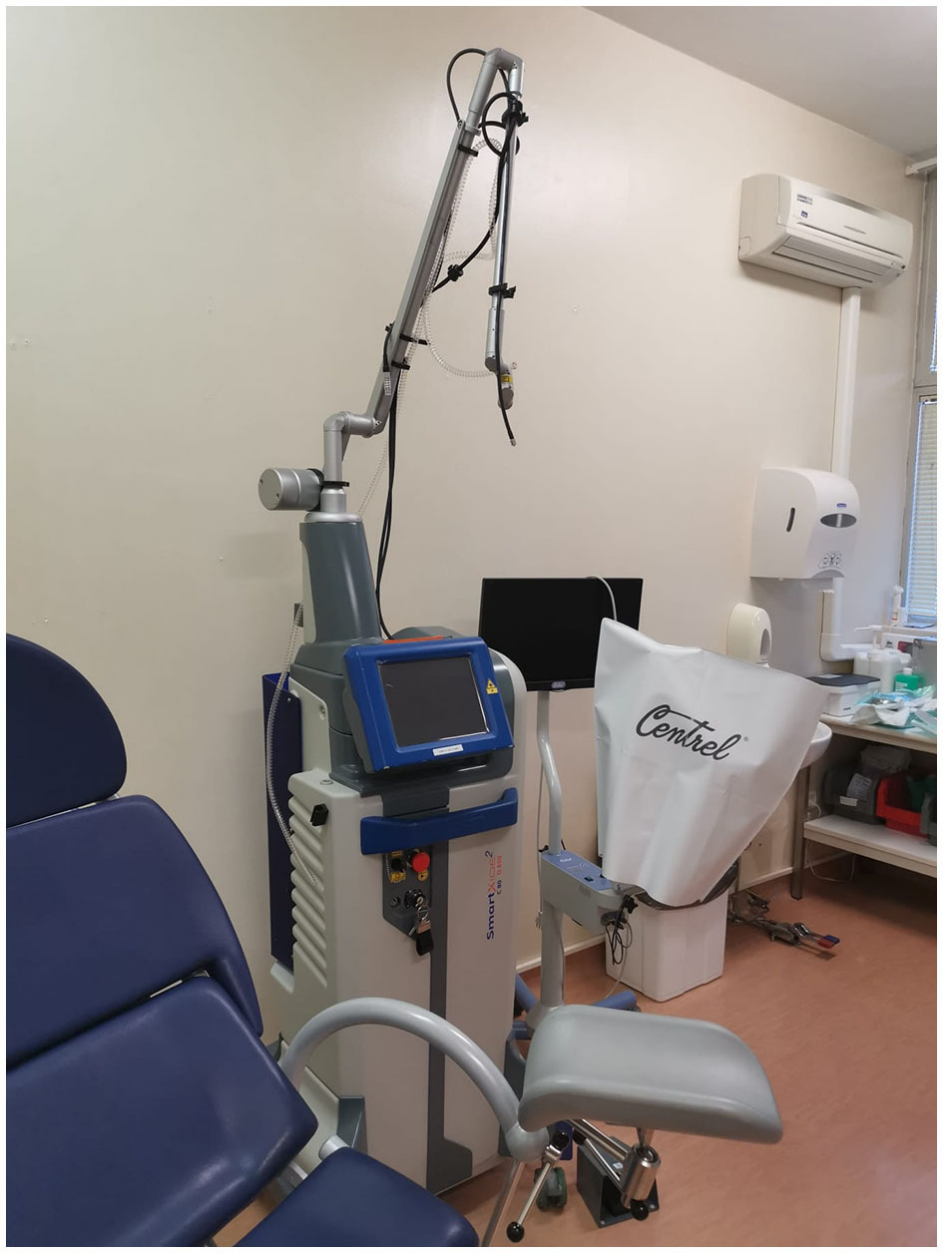
Outpatient setting: SmartXide2 C80 laser system.

System used SmartXide2 C80 laser system by DEKA, Calenzano, Italy, a RF excited CO_2_ laser, with 80 W of max power; this system is also equipped with a second 50 W 980 nm diode laser source fibre delivery, which results very useful in those procedures where higher coagulative power is necessary (Figure 1).

Accessories: scanning micromanipulator Easyspot + HiScan Surgical or ColpoScan (connected to a 300 mm lens colposcope (Z4—Centrel S.r.l.), EndoScan and microscan scanners connected to long focal 5” handpieces.

The scanner-assisted CO_2_ laser provided with the appropriate accessories was used to treat various proctologic pathologies, such as abscesses, fistulas, condylomas, AIN, as the CO_2_ laser scanning fast vaporisation and excision are extremely effective on soft tissue surgery. The article's aim is nevertheless to focus on the fissurectomy procedure.

The procedure involves two surgical stages: vaporisation and superficial vaporisation and debridement.

The CO_2_ laser was used in fissurectomy to vaporise sentinel skin tag, sentinel polyp, and fissure margins.

Here, the parameters for vaporisation used according to the scanning shape and depending to the area to treat: Clover (interpolated double ellipsoid): UP Mode, 15–20 W, dwell time 0.2 ms, continuous or repeated scanning (T-Off 0.1 s); hexagon: CW Mode, 18–25 W, dwell time 0.1 msec, repeated scanning (T-Off 0.1 s).

Then, fissure superficial vaporisation and debridement, to obtain a more uniform plane eventually removing biofilm and stimulate the tissue to regenerate, was performed using these parameters: Clover (interpolated double ellipsoid): UP Mode, 4–8 W, dwell time 0.2 ms, continuous or repeated scanning (T-Off 0.1 sec); hexagon: CW Mode, 12–18 W, dwell time 0.1 ms, repeated scanning (T-Off 0.1 s).

In case coagulation was needed, the laser emission mode was switched to CW and the power reduced.

Haemostasis, when needed, was achieved by defocusing the CO_2_ laser (Clover, low power CW mode continuous scanning), or using a monopolar electrosurgical energy or by suture.

**Figure 2:**
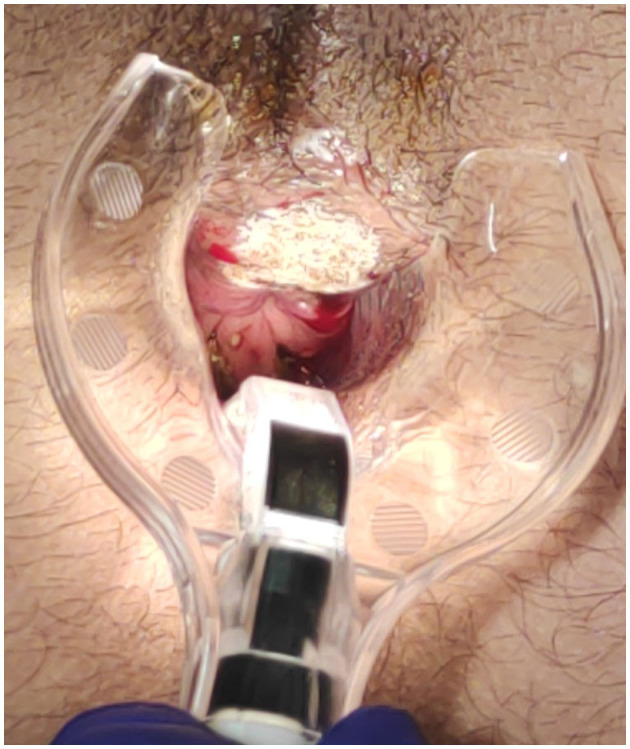
Anterior fissure: sentinel sking tag CO_2_ laser scanner vaporisation.

**Figure 3:**
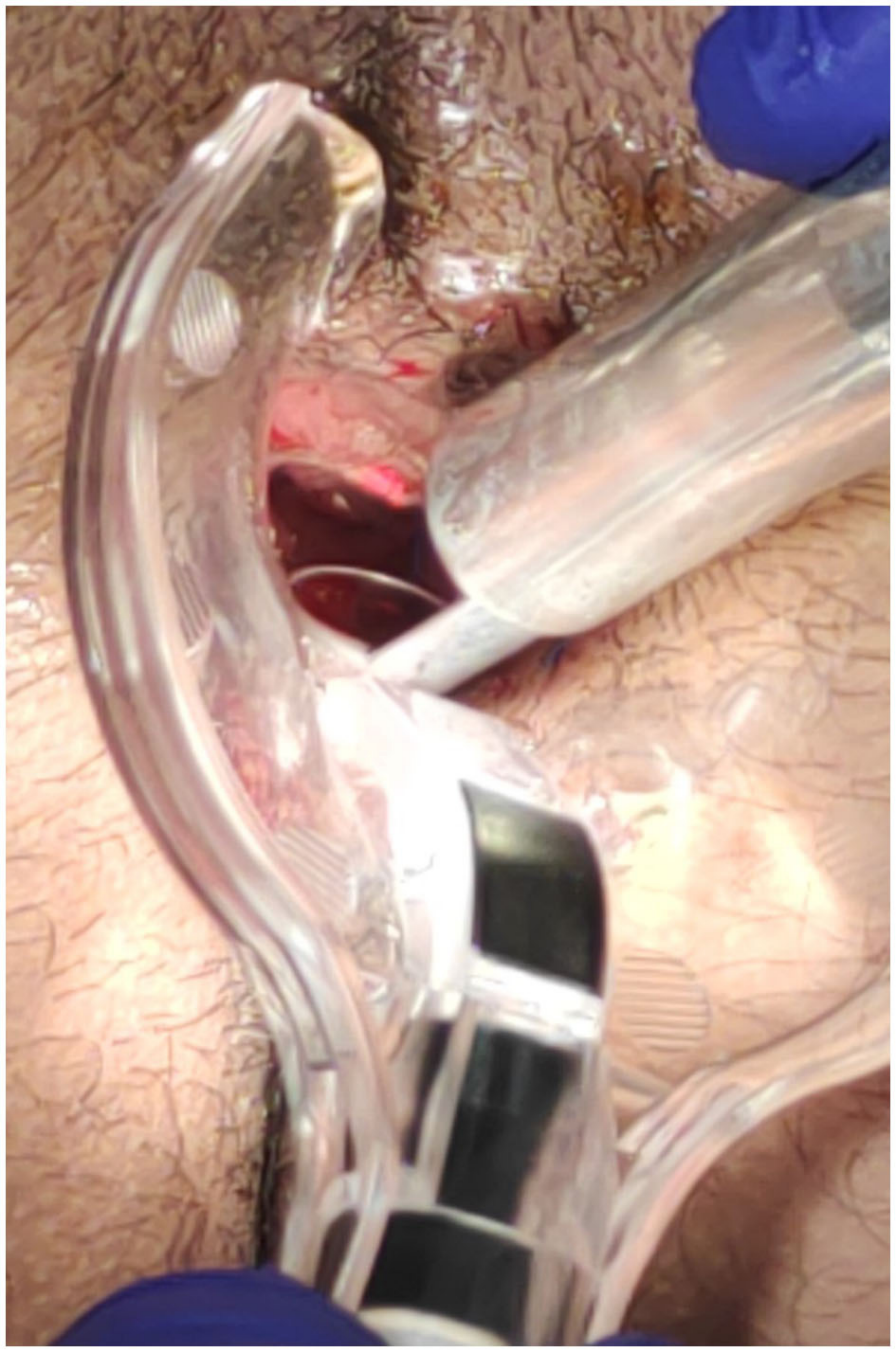
Anterior fissure: fissure CO_2_ laser scanner superficial vaporisation and debridement.

**Figure 4:**
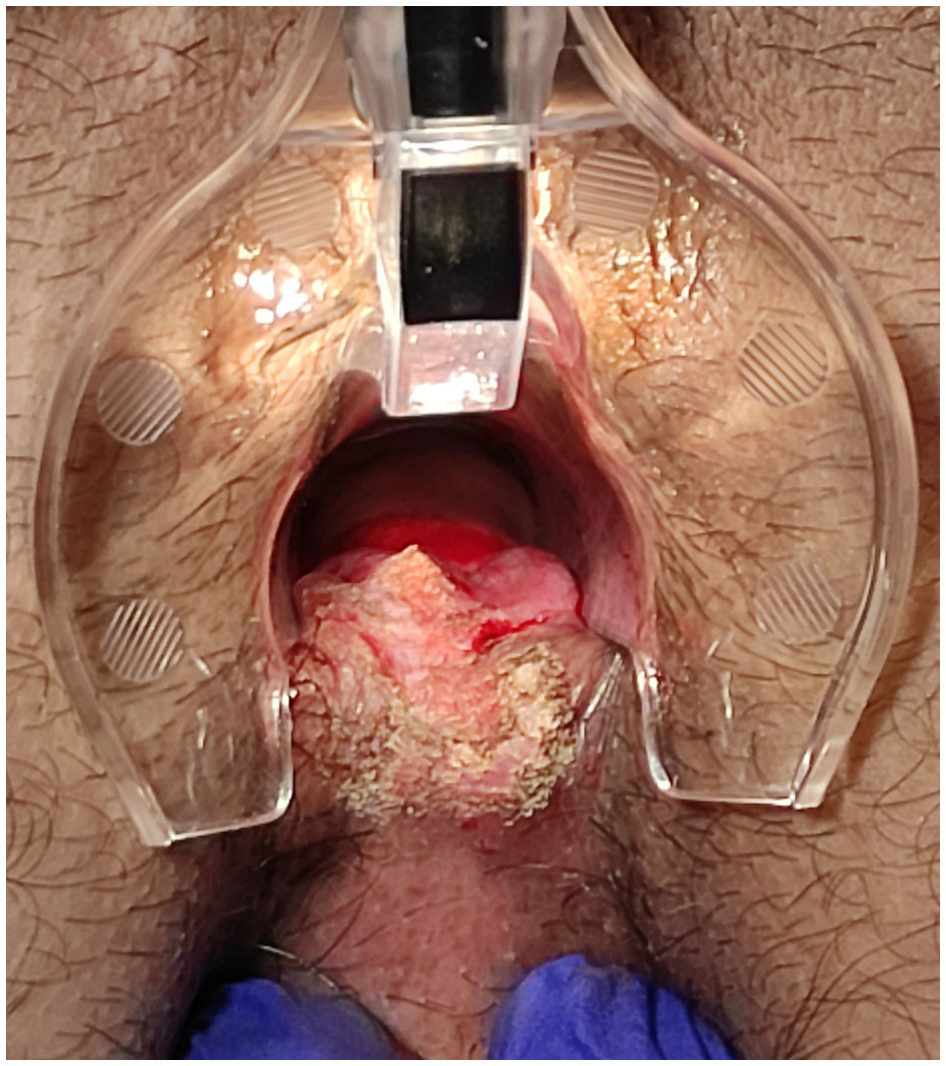
Posterior fissure: sentinel skin tag CO_2_ laser scanner vaporisation and fissure superficial vaporisation and debridement.

**Figure 5:**
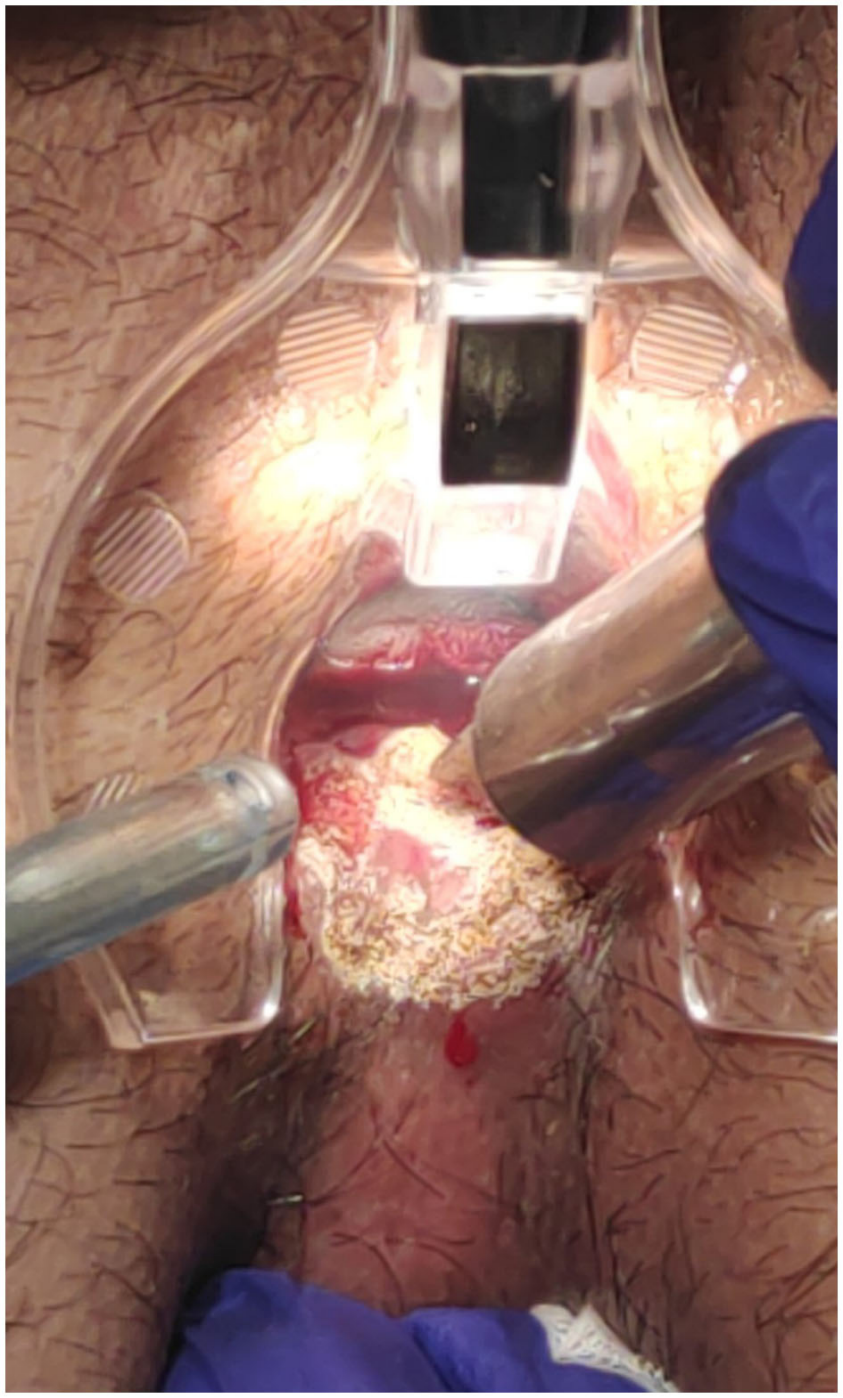
Posterior fissure: ongoing CO_2_ laser vaporisation.

The surgical goal is to achieve a smooth and dried wound with a minimal tissue thermal damage, to ensure good postsurgical pain control, rapid and functional, elastic and stable healing, and to prevent potential relapses (Figures 2–5).

All surgical data were recorded: from the number of vaporised sentinel polyp to the number of skin tag treated. Surgical time, intraoperative, 24 h, 1 week, and 1-month complications and surgeon's satisfaction (VAS 0–10) were recorded too.

### Statistical Analysis

In this study, the statistical analyses focused on postoperative results related to specific scanner-assisted CO_2_ laser treatment in a selected patient group: the clinical and follow-up data were stored in a prospective maintained database.

Association between median VAS after surgery, time of treatment after diagnosis and grade of reepithelialisation variables, patients' characteristics, and surgical procedure were examined by chi-square and Fisher's exact tests. *p-value* < 0.05 was considered significant.

XLSTAT software (version 2021.3.1) (By Addinsoft PARIS, France, Europe) was used for statistical analysis: *p* < 0.05 was considered significant.

This was a retrospective single centre study and is reported according to the Strengthening the Reporting of Observational Studies in Epidemiology (STROBE) statement for cohort studies (23).

## Results

During the study conduct period, we subjected 105 patients to proctology surgery using a scanner-assisted CO_2_ laser, as reported in Table 2.

**Table 2:** Scanner CO_2_ laser proctological procedures.

**Surgery**	**Number of patients**
Fissurectomy	29
Fissurectomy + other treatments	39
Fistulotomy	5
Treatment of fistula tract ad internal orifice	14
Hemorrhoidectomy	8
HPV Lesions vaporisation	6
Sinus Pilonidalis wound defect	3
Anal Polyp vaporisation	1
	105

In total, 29 out of the 105 patients were those who met the inclusion and exclusion criteria of the study. The mean age of these patients was 49.7 years old (range 19–84). Totally, 6 of them were women, and 3 patients out of 29 was suffering from an anterior fissure whereas 26 out of 29 from a posterior fissure. The number of sentinel skin tag was 23 whereas the number of sentinel anal polyp was 9.

In summary, the number of fissurectomy procedures alone was found to be 5 whereas the number of associate surgical procedure, for example, fissurectomy with simultaneous polyp vaporisation and/or sentinel skin tag vaporisation, was 24.

Recorded fissure and fissurectomy-related symptoms are reported in Table 3.

**Table 3:** Symptoms (number of patients) changes.

	**T0** **pre operative**	**T1** **24 h**	**T2** **1 week**	**T3** **1 month**	***p***
Pain	29	22	11	5	0.002
Bleeding	17	9	0	2	0.21
Anal itching	6	1	0	0	0.04
Burning	14	20	14	7	0.15

Overall, 17 patients reported a preoperative history of constipation in particular with episodes of hard stool and 2 of diarrhoea. Only one patient reported anal sex.

During the period of examination, 1 month after surgery, 3 patients experienced episodes of constipation, none of diarrhoea, and 1 felt confident to restart anal intercourse.

The duration of symptoms mean time was 10.6 months (range 3–36).

REALISE score was employed to evaluate and asses the severity on anal fissures. Data are reported in Table 4.

**Table 4:** REALISE score.

	**T0** **pre operative**	**T2** **1 week**	**T3** **1 month**	***p***
REALISE score	13.62 (range 7–22)	6.69 (range 4–18)	5.41 (range 4–14)	0.484

Pain severity was carefully evaluated and recorded (Table 5).

**Table 5:** Pain.

	**T0** **pre operative**	**T1** **24 h**	**T2** **1 week**	**T3** **1 month**
Mean Pain (VAS 0–10)	5.24 0 pts without pain 3 pts with pain = 3	4.45 2 pts without pain 6 pts with pain ≤3	1.96 13 pts without pain 21 pts with pain ≤3	0.86 20 pts without pain 26 pts with pain ≤3
Maximum Pain (VAS 0–10)	9.03 all with pain ≥7	7.10 1 pts without maximum pain	3.55 12 pts without maximum pain	1.59 20 pts without maximum pain
Maximum pain duration 1-With 10 m 2-Between 10 and 30 m 3-Between 30 and 60 m 4-More then 60 m	6 pts 12 pts 4 pts 7 pts	26 pts 3 pts 0 pts 0 pts	12 without pain 12 pts 3 pts 1 pt 1 pts	22 without pain 5 pts 1 pt 1 pt None
Anal digital exploration 0-not painful 1-painful 2-impossible	6 20 3	– – –	17 12 0	21 8 0
Painkiller days	–	1	4.48 11 pts still under painkiller	8.7 5 pts still under painkiller

Mean pain intensity ≤3, considered as the principal endpoint, was recorded in 26 out of the 29 patients who enrolled in the study with a final success rate of 89.7% at 1-month follow-up. This percentage reached 85.7% in our cases only 1 week after procedure.

Compliance with anal cream application was evaluated questioning each individual patient on the correct and daily application: at 1 week, 22 out of 29 responded positively whereas only 20 out of 29 at 1-month follow-up.

Degree of reepithelisation of the postsurgical fissure was measured (Table 6).

**Table 6:** Degree of reepithelisation.

**Degree**	**T2** **24 h**	**T3** **1 week**	**T4** **1 month**
0-deep fissure still present	29	0	0
1-superficial fissure	0	13	2
2-partial reepithelisation	0	15	5
3-complete healing and reepithelisation	0	1	22

No statistical correlations were found between grade of reepithelialisation after 1 month and age (*p* < 0.855), gender (*p* < 0.568), operative time (*p* < 0.506), and associate surgical procedure group (*p* < 0.258).

A most important relief, in our data, was the excellent clinical result in term of reepithelisation grade 3 in the subgroup of patient's compliant with the application of anal cream (*p* < 0.017).

An interesting correlation, even without statistical value (*p* < 0.258), was found in our data between the precocity of treatment, related to pain symptom duration before laser treatment, and reepithelisation grade. The mean duration of pain before treatment in our data was 10.6 months.

Those treated before the mean time of 10.6 months showed a very good result in term of success (grade 3 reepithelisation) (86.7%) vs. patients treated after 10.6 month (64.2%).

A correlation was also sought between the trend of mean pain intensity and reepithelisation but it was not found.

On average, the surgical procedure lasted 19.3 min (range 10–40).

### Complications

We must also report that no changes in surgical strategy have been recorded, confirming the efficacy of the anaesthetic technique, selection of patients, accuracy of the care pathway and, last but not least, the safety and reproducibility of the scanner-assisted CO_2_ laser surgery.

The recorded complications were 3 at 1 week of follow-up, all related to the painful oedema of the surgical wound margins, whereas only 1 case continued to have oedema 1 month after surgery.

No reoperation during the follow-up period was performed. Any patients complained about of faecal incontinence of all grades.

### Satisfaction

The satisfaction of the treated patients was 8.83 (VAS 0–10) at 1 week and 9.17 at 1 month. The 3 cases of failure, according to our protocol (mean pain severity scored ≤3), recorded scores 5, 7, and 7 again, respectively. Surgeons' satisfaction that was measured at the end of the surgical procedure was 9.07 (range 7–10).

## Discussion

Anal surgery is taxed by a high rate of postoperative pain. Anal fissure stigmata symptom is high intensity and long duration pain, so long that it is disabling for patients.

Most patients with anal fissure report a good response to medical therapy in terms of both pain control and healing (87%) (24); however, both are achieved very slowly, so much so that patients need several days to regain some well-being. Pain control always coincides with adequate healing. The effectiveness of medical therapies is also further lower in case of chronic fissure (50%) (25–29).

When none of the medical therapies adopted are effective, excluding the possible presence of a neoplasm through the execution of a biopsy, mandatory in doubtful cases, it is necessary to proceed with surgery. Among the numerous surgical operations proposed, from anal stretch to standardise anal dilatation with pressurised balloons to anoplasty, internal sphincterotomy has been found to be the “gold standard”.

In a Cochrane review by Nelson et al. (27), a prospective randomised controlled trial demonstrated that the internal sphincterotomy operation is more effective than medical treatments.

Though its extreme efficacy in controlling anal pain derived from the spasm of the internal anal sphincter and its capability to heal the fissure have widely been proved, it is necessary to critically analyse the result of the internal sphincterotomy which has a high rate in various degrees incontinence.

Therefore, the problem we had to face was to find a surgical solution for those patients who failed in medical therapies, a surgical solution that had to be able to heal the fissure without postoperative burden and faecal incontinence.

According to experience, the majority of patients (89.7%) reached the main goal of mean intensity pain ≤3 (VAS 0–10) 1 month after the operation. Moreover, this percentage had already reached optimal outcomes (85.7%) 1 week after procedure: clear evidence of high value in terms of both absolute effectiveness and speed. The maximum pain intensity disappeared in 68% of our cases after 1 month although 5 patients started from a value of VAS ≥ 7. The initial mean value was equal to 9.03 whereas it was equal to 1.59 after 1 month.

This result is comparable with reports in the literature of internal sphincterotomy surgery (26).

Moreover, it is interesting to notice the healing rate speed of this approach compared with traditional fissurectomy (30).

However, our aim was to analyse the symptom pain from different points of view, as suggested by other authors (16).

Maximum pain intensity at 1 month disappeared in 20 out of 29 patients, 5 of them had a VAS ≥7. The initial mean value was equal to 9.03 whereas it was equal to 1.59 after 1 month.

In particular, we measured the duration of the maximum perceived pain, an important consequent element of disability but often little considered in the literature: 1 month after treatment, 1 patient reported a duration of pain between 30 and 60 min, 1 patient a duration between 10 and 30 min whereas 5 patients <10 min.

Anal digital exploration by the surgeon was evaluated and was measured at both 1-week and 1-month follow-up, resulting in zero-not painful, respectively, in 17 and 21 out of the 29 patients who were examined. This report has the function of objectifying the patient's pain during the visit and associating it with what was reported during the collection of the medical history.

Digital exploration also has the function of monitoring the healing process and adopting any changes in the postsurgical medical strategy, from the indication to the use of other creams (29, 31, 32) to the use of anal dilators (33).

Stepping back to the symptom pain, we have also investigated the pain impact on the daily patients' life by measuring how long was the painkiller self-intake period: at 1 week, the average number of days reached a value of 4.48 and 11 out of 29 patients reported that they continue to intake painkillers because of the need; at 1 month, the mean reached a value of 8.7 days with only 5 patients continuing to take painkillers.

This result indirectly indicates how much the pain weakens each patient and represents an objective element of analysis that allows to homogenise the subjective variables of each treated case.

Since the painkiller days was taken, value of 8.7 shows that particular attention has to be paid to the first week; moreover, any strategies to be adopted in addition to those explored, which may allow a reduction in this value, should be evaluated.

Anyway, we never forget that the anal fissure can be characterised not only by the pain symptom but also by other symptoms that can be equally weakening and that we therefore wanted to analyse bleeding, anal itching, and burning (16).

Our experience demonstrates that the strategy adopted is able to determine a significant reduction primarily not only in pain but also in the other 3 symptoms too. In particular, the result on burning, however, deserves to be analysed: though surgery halves the symptoms, 7 patients still continue to suffer from it 1 month after and in any case present together with pain in the 3 patients with mean VAS ≥ 3.

The recent introduction in the literature of a score dedicated to the clinical evaluation of the anal fissure, or the REALISE score (14) allowed us to adopt it in our series: we were able to record a statistically significant reduction between the situation before surgery and the follow-ups at 1 week and at 1 month, since it confirms the great benefits and efficacy of the surgical strategy adopted.

Despite the low number of failure cases, we still wanted to evaluate what the variables affecting the outcome might be.

The statistically significant correlation between adherence to the anal cream application and healing demonstrated, in our experience, the importance of this local postoperative procedure: clearly emerged from the literature and also the need to continue with the suggested treatment for at least 6 weeks (29).

These data are essential to better educate patients in postsurgical wound care. Our mind to allow functional healing is to daily treat these wounds by applying a cream with a finger, as claimed by other authors (34).

About reepithelisation our experience shows an important correlation with adherence to the anal cream application and no correlation with other variables.

An important relief was the relationship with symptoms' duration: those patients with a shorter symptom duration actually demonstrated a better healing tendency than the others, suggesting the early use of fissurectomy.

In addition, the lack of correlation between pain resolution and level of healing opens up an interesting discussion and also confirming what the literature has already demonstrated. In fact, it had already emerged that the pain had a faster resolution than reepithelialisation, a dynamic that must be known by an expert and essential to proctologist to better follow the healing process (30).

Looking at the pure numbers, we would like to underline that 27 out of 29 patients demonstrated complete (22) or partial (5) reepithelisation.

This result is extremely superior to the reports in the literature regarding traditional diathermy fissurectomy, which averages a mean healing time of 10.3 weeks (30). Although our experience involves a small group of patients, the results obtained are so clear that they represent a solid basis for future studies.

CO_2_ laser technology, widely used in several surgical fields (otorhinolaryngology, neurosurgery, wound healing, and gynaecology) for many years, has enjoyed a technological upgrade thanks to the introduction of scanning units that have allowed the execution of extremely delicate, precise, and effective procedures.

By analysing the literature about the use of laser technology in fissurectomy operations, we found that in 2015, an Iranian group had explored the use of CO2 laser for fissurectomy and fractional CO_2_ laser for a multipoints myotomy of the internal anal sphincter (as an alternative to internal sphincterotomy), demonstrating an extremely rapid healing with no relapses at 1 year of follow-up (35).

Scanner-assisted CO_2_ laser has also been used to promote secondary intention healing of several wounds: first of all, diabetic foot ulcers through bacterial load reduction and the promotion of healing (36, 37); also in sporadic cases of complex wounds such as a rectal vaginal fistula in a patient with Crohn's disease (38).

In our experience where this technology has been used for the very first time in proctology surgery, the scanner-assisted CO_2_ laser demonstrated to be extremely safe and effective as no cases of change of strategy during surgery or complications related to its use have been reported. It has also proved to be an excellent ally in settings such as the outpatient one in the context of a less invasive care pathway also thanks to the use of local anaesthesia and careful selection of patients. Moreover, the same scanner can also be used in the fractional mode to stimulate the tissue regeneration.

The new technical solutions introduced with this new generation of CO_2_ lasers are paving the way to new and fascinating scenarios of use that will be the subject of future studies and that are radically changing the surgical approach to proctologic pathologies.

Let us now analyse the impact of the intervention we proposed on faecal incontinence: no patient complained of any episode or degree of incontinence after 1 month of follow-up.

We therefore asked ourselves whether or not it is right to continue to argue that internal sphincterotomy is considered the gold standard in the treatment of anal fissure. It would probably be more correct to proceed with a less invasive method such as scanner-assisted CO_2_ laser fissurectomy and restrict the sphincterotomy to those cases complaining of pain persistence during the follow-up: this tailored attitude could drastically reduce the number of cases of incontinence while increase the percentage of healed and satisfied patients.

However, few said about any relapses as our experience is limited to only 1 month of follow-up. The certainty is that relapse prevention is the result of a series of elements that must act synergistically with each other: first of all, a functional, elastic, and stable postfissurectomy healing, the correction of constipation or the use of enemas in case of hard stools, the application of an anal cream in case of diarrhoea, and a careful surgical follow-up that can identify any delays or defects in healing that require changes in the medical strategy (other creams, dilatant, etc.).

### Limits of the Study

This study reports the very first application of a scanner-assisted CO_2_ laser technology in proctologic surgery and, more specifically, in the treatment of chronic anal fissure through a fissurectomy procedure.

The limits this procedure presents are related to the small number of patients treated in addition to the fact that it was conducted in a single centre, without randomisation too.

The excellent outcomes obtained are however an excellent basis to develop future experiences.

It will also be essential to explore the possibility of carrying out this procedure in the context of enhanced recovery after surgery programmes and possibly in association with different anaesthetics or analgesics.

In future, it will be necessary to design multicentre randomised trials that can confirm the results obtained either in terms of fissure healing, low complications (as faecal incontinence), and postoperative pain, studies that can make use of new digital technologies such as telemedicine (39) or the use of digital custom-made applications to follow patients.

## Conclusions

Scanner-assisted CO_2_ laser turned out to be a useful aid in proctology surgery and in particular during fissurectomy, as it allows extremely precise ablation, vaporisation, and debridement procedures with minimal lateral thermal spread.

The wide versatility of pulse shape and energy delivery has proved to be both an added value by allowing an extreme modulation of the energy delivered but also a limitation due to the lack of consolidated experience with the use of this technology in this surgical field.

Randomized multicentre studies based on comparison with traditional techniques are the next step necessary to ensure the good results obtained with this experience.

## Data Availability Statement

The original contributions presented in the study are included in the article/Supplementary Material, further inquiries can be directed to the corresponding author/s.

## Author Contributions

IG: surgical procedures, data collection, and paper writer. TC: surgical procedures, data collection, and statistics. CL, FC, PD, GR, and CT: surgical procedures and data collection. GT: paper writer. FB, AA, SG, and AP: data collection. LG: laser supervisor and paper revisor. CE: study organizer, surgical procedure, and data collection. All authors contributed to the article and approved the submitted version.

## Conflict of Interest

The authors declare that the research was conducted in the absence of any commercial or financial relationships that could be construed as a potential conflict of interest.

## Publisher's Note

All claims expressed in this article are solely those of the authors and do not necessarily represent those of their affiliated organizations, or those of the publisher, the editors and the reviewers. Any product that may be evaluated in this article, or claim that may be made by its manufacturer, is not guaranteed or endorsed by the publisher.
